# Association Between Body Self-Perception and the Incidence of Hypertension: The SUN “Seguimiento Universidad de Navarra” Cohort 1999–2022

**DOI:** 10.3390/biomedicines13051147

**Published:** 2025-05-09

**Authors:** Patricia Caro, Carmen De La Fuente-Arrillaga, Vanessa Bullón-Vela, Rafael Pérez-Araluce, Miguel Ángel Martínez-González, Maira Bes-Rastrollo

**Affiliations:** 1 Department of Health and Wellness, University Catholic of Uruguay, Montevideo 11600, Uruguay; patricia.caro@ucu.edu.uy; 2 Public Health Institute, Universidad Andrés Bello, Santiago 7591538, Chile; 3 Department of Preventive Medicine and Public Health, University of Navarra-Instituto de Investigación Sanitaria de Navarra (IdiSNA), 31008 Pamplona, Spain; 4 CIBERobn, 28029 Madrid, Spain; 5 Department of Nutrition, Harvard T. H. Chan School of Public Health, Boston, MA 02115, USA

**Keywords:** body self-perception, hypertension, body image

## Abstract

**Objective**: This study aims to analyze the association between self-perceived body image and the incidence of hypertension. **Methods**: A prospective cohort study was conducted, classifying body image perception into three categories: adequate, underestimation, and overestimation, based on Stunkard’s Figure Rating Scale and self-reported nutritional status. Cox proportional hazards models were used to determine the association between body image perception and the risk of developing hypertension, adjusting for potential confounders. **Results**: During a mean follow-up period of 12.7 years, 2359 incident cases of hypertension were identified. In the main adjusted model, body image underestimation was significantly associated with an increased risk of hypertension among women (HR 1.25; 95% CI 1.01–1.55). This association lost statistical significance when adjusting for baseline BMI in the sensitivity analysis. **Conclusions**: Self-perception of body image may influence health behaviors that impact weight control, potentially leading to higher BMI and, consequently, greater cardiometabolic risk. Although further research is needed to clarify its role, body image perception should begin to be considered in clinical practice as a relevant factor in chronic disease prevention.

## 1. Introduction

Body image is a multidimensional construct that incorporates subjective, behavioral, and feeling processes, which can be either positive or negative regarding one’s body image [1,2]. It is the body representation of people influenced by culture and social values [3].

The way people see and feel about their bodies is called body image perception and constitutes a mental representation of their appearance [4]. Therefore, the disparity between actual body image and one’s perception is called body dissatisfaction [5]. Sociodemographic, psychosocial, and lifestyle factors have been associated with body dissatisfaction, such as being a woman, an adolescent, white, exposed to bullying, obese, and sedentary [6,7].

There are different ways to evaluate body image perception [8], one of which is through silhouette tests [9]. According to the silhouette test, people can classify themselves as underweight, accurate weight, overweight, or obese. When compared with their nutritional status according to BMI, these classifications can lead to accurate, underestimated, or overestimated perceptions of their nutritional status.

A close relationship has been observed between body dissatisfaction and eating disorders, mental health outcomes, and unhealthy weight control practices in teenagers [10,11] and young adults [12,13,14,15,16], but there is a lack of evidence linking body dissatisfaction and health outcomes in adults.

Obesity is a public health challenge, recognized as a principal contributor to chronic diseases and a major factor in the global health burden [17]. Hypertension is recognized as the leading preventable risk factor for premature death and disability [18], responsible for at least 53% of deaths from heart disease and 53% of deaths from stroke. Evidence has shown that obesity is a causal factor of hypertension [19,20], and body image can be related to obesity and the practices for weight control that people adopt.

The mechanism that could explain this relationship is based on social experiences and the psychological stress that being obese represents. Currently, in Western societies, thinness is idealized more strongly among women, which causes them to feel more dissatisfied with their body image compared to men, tending to overestimate their body image more frequently; that is, they perceive themselves as having a worse nutritional status than they actually have. Among men, the idealized image is that of a muscular man with a large body; for this reason, men tend to underestimate their body image; that is, they see themselves with a better nutritional status [21,22].

The evidence showed a relationship between negative perceptions of body image, poor eating habits, and sedentary lifestyle, in addition to depression and anxiety [14]. These conditions do not contribute to weight loss and increase the risk of high blood pressure. Given the relationships that have been evidenced between body image perception, obesity, and other health outcomes, understanding how self-perception of body weight influences the risk of hypertension becomes increasingly relevant. This research aims to investigate the association between self-perception of body image and the incidence of hypertension and the modifying effect that sex may have, in a well-established Mediterranean cohort.

## 2. Materials and Methods

### 2.1. Study Population

The “Seguimiento Universidad de Navarra” (SUN) project is a cohort, prospective, and dynamic study conducted among Spanish university graduates. Its main objective is to determine the relationships between various lifestyle and dietary patterns and the incidence of several chronic illnesses, such as hypertension. The methodological framework, including recruitment and design aspects of the SUN project, has been previously reported in studies [23]. Participant recruitment started in December 1999. Once they had completed the validated baseline questionnaire, participants were subsequently contacted every two years via e-mail or mail to complete a follow-up questionnaire. As of 31 May 2022, 23,133 individuals had completed the baseline questionnaire.

**Figure 1:**
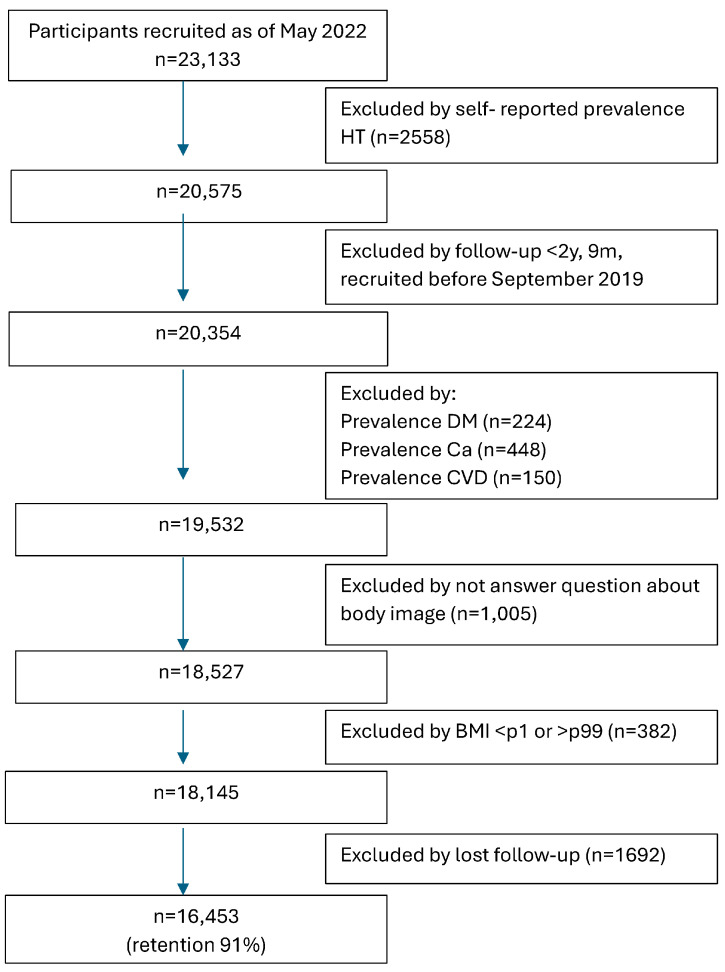
Flowchart of participant inclusion in the SUN Cohort, Spain 1999–2022. HT = Hypertension.

For this analysis, we excluded 2558 individuals who had previously been diagnosed with hypertension (self-reported, physician-diagnosed hypertension; anti-hypertensive medication use; or self-reported systolic blood pressure (SBP) > 140 mmHg or diastolic blood pressure (DBP) > 90 mmHg) at the time of enrollment. Only participants who had completed the baseline questionnaire before 31 September 2019 (*n* = 20,354) were considered, ensuring a minimum follow-up period of 2 years and 9 months to allow adequate time to complete the baseline questionnaire and minimize the risk of selection bias. Additionally, 822 participants with prevalent chronic diseases at baseline (including diabetes, cancer, and cardiovascular disease), 1005 participants who did not answer the body self-perception image question, 382 participants with weight measurements outside the first or ninety-ninth percentiles, and 1692 individuals lost to follow-up were excluded. Our final analysis included 16,453 participants with 2359 incident cases of hypertension. The overall long-term retention rate for the cohort was 91% (Figure 1).

### 2.2. Exposure Variable: Body Self-Perception

Body self-perception was assessed using Stunkard’s Figure Rating Scale (FRS). The FRS is widely recognized as a valid and reliable tool for the evaluation of the body self-perception in a diverse population, including both women and men [24,25]. In the baseline questionnaire, participants were asked the following question: “Mark the silhouette which is the most similar to yours at this moment” [24,25].

The body mass index (BMI, kg/mt^2^) was calculated using the participant’s self-reported weight and height at baseline to determine nutritional status. The accuracy of self-reported weight and height was previously validated in a subsample of the cohort, obtaining a kappa index of 0.91 (95% CI: 0.81–0.99) and a correlation coefficient of 0.991 (95% CI: 0.986–0.994) between self-reported and measured weight [26].

Then, three categories were created using the self-reported nutritional status and body self-perception categories as follows:

1. Adequate: Defined as when the nutritional status perceived through FRS matches the nutritional status determined by BMI.
2. Underestimation: Defined as when the nutritional status perceived through FRS was lower than the nutritional status determined by BMI.
3. Overestimation: Defined as when the nutritional status perceived through FRS was higher than the nutritional status determined by BMI.

### 2.3. Outcome Variable: Hypertension

The principal outcome variable was the incidence of self-reported hypertension. The baseline and follow-up questionnaires asked participants whether they had ever been diagnosed with hypertension and recorded their systolic blood pressure (SBP) and diastolic blood pressure (DBP) measurements. Participants were also asked if they were taking any anti-hypertensive medications. Incident hypertension was defined as an SBP of at least 140 mmHg and a DBP of at least 90 mmHg and/or taking antihypertensive medications, following the criteria outlined in the 8th Report of the Joint National Committee [27]. A previous sub-sample for this cohort study confirmed the reliability of self-reported hypertension diagnoses. This study demonstrated that 82.3% (95% CI: 72.8–92.8) of participants who reported having hypertension were confirmed by standard blood pressure measurement, and 85.4% (95% CI: 72.4–89.1) of those who did not report hypertension were confirmed as non-hypertensive [28].

### 2.4. Other Covariates

Data collection was conducted using previously validated questionnaires, which included information on socio-demographic characteristics (age and sex) and anthropometric variables (height and weight) [26]. Average weight during the follow-up was estimated, and participants were asked about health-related behavior, such as smoking status (never, current, or former smoker), alcohol consumption (g/day), physical activity (METs-h/week) [29], and dietary intake asses through a validated food frequency questionnaire. Based on dietary data, total energy intake (kcal/day), sodium and potassium intake (mg/d), adherence to the Mediterranean dietary pattern (low (0–3)), moderate (4–6), high (7–9), and the intake of ultra-processed food (serving/day) were calculated. Additionally, the questionnaire collected information on family and personal history at baseline, including diagnoses of hypercholesterolemia, hypertriglyceridemia, and depression, as well as the use of analgesic medications. Adherence to the Mediterranean diet (MedDiet) was evaluated using a score based on nine items proposed by Trichopoulou et al. [30]. The reliability of self-reported data has been previously validated in earlier studies [29,31].

These variables were included as potential confounders to reduce the risk of bias in estimates of the association between body image perception and incidence of hypertension based on biological plausibility and empirical evidence.

### 2.5. Statistical Analysis

Participants’ baseline characteristics were described using descriptive statistics. Continuous variables were reported as means with standard deviations for normally distributed data and medians and interquartile ranges for non-normally distributed data. The Shapiro–Wilk test was employed to assess the normality of distribution. Categorical variables were expressed as absolute frequency and proportions. Follow-up time was defined from the date of completion of the baseline questionnaire to either the date of the last follow-up questionnaire or the date of self-reported hypertension diagnosis (incident case), whichever occurred first.

To determine the association between body self-perception and the risk of hypertension, we used the Cox proportional hazards model. This approach allows for the estimation of time-to-event data, enabling us to evaluate how body self-perception at baseline influences the risk of hypertension over time. On the other hand, using body self-perception measured at baseline complies with the model’s assumption that covariates remain constant throughout the risk period. Hazard ratios (HR) and their corresponding 95% confidence interval (CIs) were calculated. Age was used as the underlying time variable in the analysis, and the ‘adequate perception’ category was set as the reference group.

Firstly, it was verified that the risks were proportional over time. We ran an age- and sex-adjusted model and a multivariable model, adjusting for possible confounders to describe in the other covariate section. Missing data was handled by simple imputation, using logistic or multinomial regression for categorical variables. We imputed the following variables: following a specific diet (2.5%) and weight change (2.9%).

In addition, we ran the multivariable model by adding adjustments by categories of transition of underestimation (actual status of obesity and perceived overweight; actual status of overweight and perceived normal status; actual status of normal and perceived underweight) or overestimation (actual status of normal and perceived overweight, or actual status of overweight and perceived obesity). In this analysis, categories with fewer than 15 data points were excluded.

The incorporated BMI during follow-up or obesity status in the multivariable model resulted in multicollinearity, likely due to their strong correlation with body self-perception. To address this, we replaced these variables with participants’ average weight during follow-up, which served as a proxy for body mass without compromising model stability.

Several sensitivity analyses were performed to estimate the fully adjusted hazard ratios, specifically examining the association between underestimation and the onset of hypertension, using the adequate perception group as a reference. Data analysis was conducted using STATA version 14.0 (StataCorp, College Station, TX, USA). We defined statistical significance as a *p*-value < 0.05.

## 3. Results

The analysis included 16,453 participants (10,625 women and 5828 men) with no prior diagnosis of hypertension, a mean age of 35.9 years (SD: 10.9), and a mean BMI of 23.1 kg/m^2^ (SD: 3.13).

Their total follow-up period amounted to 197,029 person-years, with a median follow-up of 12.7 years (SD: 5.4). During this time, 2359 participants developed hypertension incidents. The crude incidence of hypertension was 11.97 cases/1000 person-years (men: 19.6 cases/1000 person-years; women: 9 cases/1000 person-years). The baseline characteristics of the participants are presented in Table 1, according to categories of body self-perception and stratified by sex.

Men and women in the underestimation group were generally older and had a higher mean BMI and energy intake. They showed the highest prevalence of cardiovascular risk factors, such as hypercholesterolemia and hypertriglyceridemia, as well as a higher prevalence of hypertension in their family history. Furthermore, they reported following a special diet in a higher proportion than the adequate perception group. The men in this group reported more physical activity in leisure time than the women in the same group. On the other hand, the women in the underestimation group had a higher proportion of use of analgesics and baseline depression compared to the other groups (adequate and overestimation) and men. Other factors showed no significant differences across the groups. In the underestimation group, more than 90% were overweight or obese, and the average weight during the follow-up was higher in this group.

Most hypertension cases (*n* = 921 in men and *n* = 799 in women) were identified in the adequate perception group, with an incidence rate of 18.3 × 10^−3^ in men and 7.6 × 10^−3^ in women. However, men and women had the highest incidence in the underestimation group (26.4 × 10^−3^ and 17.4 × 10^−3^, respectively). Considering the adequate perception group as the reference, in the age and sex-adjusted model, the risk of hypertension incidence increases in the underestimation group showed HR (95%CI) 1.29 (1.13–1.49) for men and HR (95%CI) 1.92 (1.60–2.40) in women, statistically significant differences were observed only among women. The associations decreased by 23% in men and 67% in women in the multivariable-adjusted model (Table 2).

The risk of hypertension incidence according to underestimation and overestimation transitions of body image is presented in Table 3. In this table, we can see that both men (HR 1.95; 1.59–2.38) and women (HR 2.31; 1.64–3.26) who were obese according to BMI but perceived themselves to have a lower nutritional status (overweight) had a higher risk of incidence of hypertension adjusted for age and sex, this associations decreased by 74% in men and 131% in women in the multivariate model losing statistical significance. Only in the group of women who were overweight according to BMI but perceived themselves to have an adequate nutritional status HR (95% CI) 1.70 (1.32–2.18) and those who had a normal nutritional status according to BMI but perceived themselves as underweight HR (95%CI) 2.11 (1.003–4.46) presented an increase in the risk of hypertension incidence in the age- and sex-adjusted model (*p* < 0.005), in the multivariate model the risk decreased by 41% and increased 78% respectively. No significant results were observed in the transitions linked to the underestimation of the body image group among men.

Several sensitivity analyses were conducted to assess the robustness of our findings. Excluding participants under different scenarios yielded results consistent with those of the main analysis, with one exception: when baseline BMI was included in the model, we observed a loss in the statistical significance found in the group of women. This suggests that BMI plays an important mediating role in the relationship between body self-perception and hypertension risk. It reinforces the notion that inadequate body self-perception may influence BMI over time, which, in turn, is a well-established risk factor for hypertension (Table 4).

## 4. Discussion

This study has analyzed the relationship between self-perception of body image and the incidence of hypertension in university graduate adults. In the sample studied, 77% had an adequate perception of their nutritional status, which is expected since people with a higher educational level tend to be more accurate in their self-perception of weight [32]. In turn, 1 in 6 women overestimated their body image, consistent with the literature [33,34,35]. In contrast, male gender was associated with a greater frequency of underestimation of both weight status and body size. These results are in accordance with a previous study performed on a young Mexican population [34] and a European Union population [35].

A systematic review showed that those who overestimate their body weight and were seen with a nutritional status of overweight or obesity were more likely to carry out actions to reduce their weight, which can be healthy (healthy eating and physical activity) or unhealthy, associated with an increased risk of eating disorders, in turn, with a higher prevalence of mental health problems [36]. Longitudinal studies showed a more significant weight gain in length; the actions carried out were not related to better nutritional status [37].

On the other hand, the “Theory of Visual Normalization” [38] suggests that constant exposure to obesity in the environment generates a normalization of larger body size in terms of weight, which can influence people to perceive themselves as having a lower nutritional status than they have, if we add to this, the negative stigma of the diagnosis of obesity. The stress and mental health problems that can be generated in people, along with dissatisfaction with body image, could be factors that explain why people prefer to classify themselves with a healthier nutritional status [39,40].

It has been described that perceived nutritional status is a factor to be considered, as it can act as a facilitator or a limitation to initiating or maintaining weight control practices [37,41]. Research has shown that, regardless of actual weight, weight loss behaviors are more related to perceived nutritional status than to nutritional status based on BMI, with weight perception acting as a starting point for weight control or managing any weight management strategy [41]. A study performed by Hassan et al. [42] showed that participants with a misperception of weight were 85% less likely to attempt to lose weight than those with an accurate perception of their weight. Conversely, those who underestimate their body image tend to be less willing to take healthy steps to lose weight, which increases the likelihood of obesity and its cardiometabolic consequences, such as hypertension, diabetes, and dyslipidemia [43].

There is no previous evidence that links self-perception of body image with chronic diseases such as hypertension; however, in this study, it was observed that more than 90% of participants with overweight and obesity were in the underestimation group, and the average follow-up weight was higher in this group. The mechanism of action of self-perception of body image would be through implemented measures of care or carelessness of health that would promote a worse nutritional status and thus a greater predisposition to suffer from chronic non-communicable diseases. It is not possible to separate the effect of BMI and misperception about body image and their relationship with chronic diseases. BMI continues to be a more powerful predictive risk factor for chronic diseases than misperception of body image. However, the misperception of body image could contribute to people having a worse BMI. Consequently, our analysis now more clearly highlights the central and mediating role of BMI in the relationship between self-image perception and arterial hypertension, highlighting that the psychological factor of body perception can exert its impact indirectly through behavioral or physiological mechanisms that affect weight status and, consequently, increase the risk of hypertension.

### Strengths and Limitations

This study has several limitations that should be considered. The cohort predominantly includes young adults with higher educational levels, which may limit the applicability of the results to the general population. Nonetheless, this characteristic of the sample enhances the study’s internal validity, as the relatively homogeneous socioeconomic and educational profile helps reduce confounding associated with these variables. Another limitation lies in the reliance on self-reported information, such as dietary intake captured through a previously validated food frequency questionnaire, lifestyle behaviors, body weight, and hypertension diagnosis, which could introduce reporting bias. Still, the validity of these instruments has been established in previous research [29,31], which supports the consistency of the results.

Nevertheless, incorporating variables such as obesity or body mass index (BMI) during the follow-up into the multivariable model was not feasible due to issues of multicollinearity with the outcome variable. This challenge stems from the strong association between body image perception and nutritional status, particularly when body image is treated as a categorical measure. To address this limitation, we opted to use participants’ mean body weight throughout the follow-up period as a proxy indicator, which allowed us to maintain model stability while partially accounting for body composition.

On the other hand, this study also has notable strengths, such as its prospective design, dynamic cohort structure, extended follow-up period, large sample size, and excellent participant retention. A broad range of potential confounders was accounted for in the analysis, and multiple sensitivity tests were performed to ensure robustness and mitigate bias.

A wide range of potential confounding factors was accounted for, and various sensitivity analyses were conducted to enhance the robustness of our results and reduce the risk of bias.

## 5. Conclusions

Body image perception appears to play an important role in people’s health-related behaviors, potentially influencing their nutritional status and, consequently, their risk of chronic diseases. When people accurately perceive their body weight, they are more likely to adopt or maintain healthy lifestyle habits, such as a balanced diet and regular physical activity. Conversely, misperceptions, particularly underestimations, can delay the recognition of excess weight and reduce motivation to implement preventive strategies, thus contributing to an increased risk of chronic diseases such as hypertension.

While existing evidence on the relationship between body image perception and chronic diseases in adults remains limited, our findings underscore the relevance of this psychosocial factor. Further research is needed to clarify the underlying mechanisms and long-term implications. In the meantime, incorporating body image assessment into clinical history-taking can offer valuable information for the early identification of individuals at risk and for promoting personalized preventive interventions.

## Figures and Tables

**Table 1:** Baseline characteristics of participants among categories of body self-perception and sex.

Characteristics	Men (*n* = 5828)			Women (*n* = 10,625)		
	Adequate	Underestimation	Overestimation	Adequate	Underestimation	Overestimation
Total (*n*/%)	4287 (73.6%)	915 (15.7%)	626 (10.7%)	8651 (81.4)	594 (5.6%)	1380 (13%)
Age (p25; p75)	37.5	41	39.5	31.8	35.8	30.4
	(29.5; 47.5)	(32.5; 50.3)	(29.8; 49.3)	(26; 40.3)	(29.3; 45.5)	(24.8; 40)
Baseline BMI (kg/m^2^) (p25; p75)	24.5	26.3	24.2	21.4	26.3	21.3
	(22.8; 26.7)	(25.4; 30.1)	(23.4; 24.6)	(20.2; 22.9)	(25.4; 30.4)	(18.2; 23.7)
Average weight during follow-up (p25; p75)	82.8	89	80.7	63.3	77.8	61.3
	(75.2; 91.0)	(81.2; 98.0)	(74.6; 87.6)	(57.8; 70.2)	(69.4; 87.4)	(54.8; 68.6)
Overweight and Obese (%)	43.8	91.7	1.1	9.7	94.4	0.07
Weight change (%) ^1,2^						
No weight change	27.7	23.3	24.4	22.8	7.5	22.8
Weight loss	19.9	19.6	16.7	28.2	25.8	28.4
Weight gain	52.3	56.9	58.9	49.1	66.8	48.8
Smoking status (%)						
Never	48.5	44.2	47.0	52.7	49.0	51.3
Current	22.2	19.2	22.9	23.2	22.2	25.0
Former	29.3	36.6	30.1	24.1	28.8	23.7
Physical activity (METs-h/wk) (p25; p75)	21.5	18.3	17.6	14.5	11.4	12.6
	(9.4; 39.3)	(6.7; 35.9)	(7.5; 30.6)	(4.2; 27.7)	(3.0; 25.1)	(3.3; 25.8)
Energy intake (kcal/d) (p25; p75)	2495	2397	2457	2370	2312	2413
	(2009; 3058)	(1948; 2980)	(2019; 2999)	(1947; 2905)	(1894; 2858)	(1946; 2946)
Sodium intake (mg/d) (p25; p75)	3770	3772	3727	3590	3546	3640
	(2776; 5077)	(2588; 5014)	(2764; 5003)	(2736; 4741)	(2624; 4682)	(2731; 4971)
Potassium intake (mg/d) (p25; p75)	4537	4499	4463	4844	5042	4834
	(3641; 5614)	(3580; 5538)	(3634; 5682)	(3875; 6076)	(3926; 6224)	(3854; 6089)
Ultra-processed food intake (serving/d) (p25; p75)	3.5	3.4	3.5	3.2	2.9	3.3
	(2.4; 5.0)	(2.3; 4.8)	(2.3; 4.8)	(2.2; 4.5)	(2.01; 4.2)	(2.2; 4.7)
Fast food intake (serving/w) (p25; p75)	1.4	1.0	1.4	1.0	1.0	1.0
	(0.5; 2.0)	(0.5; 1.9)	(0.5; 1.9)	(0.5; 1.9)	(0.5; 1.9)	(0.5; 1.9)
Alcohol intake (g/d) (p25; p75)	6.8	7.5	6.0	2.1	1.4	2.1
	(2.1; 13.1)	(2.2; 14.9)	(2.1; 13.1)	(1.0; 5.9)	(1.0; 4.7)	(1.0; 6.0)
Trichopoulou’s Mediterranean diet score (p25; p75)	4.0	4.0	4.0	4.0	4.0	4.0
	(3.0; 5.0)	(3.0; 6.0)	(3.0; 5.0)	(3.0; 5.0)	(3.0; 6.0)	(3.0; 5.0)
Following a specific diet (%) ^2^	15.1	18.3	15.6	15.9	23.0	16.1
Family history of hypertension (%)	35.4	41.8	41.4	42.2	52.0	40.1
Prevalent Hypercholesterolemia (%)	17.6	23.4	20.9	10.6	15.5	11.6
Prevalent Hypertriglyceridemia (%)	8.4	14.5	8.5	2.2	6.1	2.0
Baseline depression (%)	2.9	3.1	3.4	4.9	6.9	6.5
Use of analgesic drugs (%)	7.8	7.4	8.8	12.1	17.5	11.6

^1^ Weight change before baseline; ^2^ Imputed values: following a specific diet (2.5%), weight change (2.9%).

**Table 2:** Hazard ratios (95% confidence intervals) for incident hypertension according to categories of body self-perception.

	Body Self-Perception		
	Adequate	Underestimation	Overestimation
**Men**			
Cases/person-years	921/50,234	259/9829	144/7495
Incident rate 10^−3^ years^−1^ (95%CI)	18.3 (17.1–19.5)	26.4 (23.3–29.7)	19.2 (16.4–22.7)
Age-adjusted model	Reference	1.29 (1.13–1.49) *	0.98 (0.82–1.17)
Multivariate-adjusted model ^a^	Reference	1.06 (0.92–1.23)	0.99 (0.83–1.18)
**Women**			
Cases/person-years	799/105,231	110/6330	119/17,052
Incident rate 10^−3^ years^−1^ (95%CI)	7.6 (7.1–8.1)	17.4 (14.4–20.9)	6.9 (5.8–8.3)
Age-adjusted model	Reference	1.92 (1.6–2.4) *	0.92 (0.76–1.12)
Multivariate-adjusted model ^a^	Reference	1.25 (1.01–1.55) *	0.92 (0.75–1.11)

* *p* < 0.005. ^a^ The main model was adjusted for several potential confounders, including age, sex, smoking status (categorized as never, current, or former), physical activity levels (measured in MET-hours per week), and participants’ average weight throughout the follow-up period. Additional adjustments included adherence to the Mediterranean diet (MedDiet score), total energy intake (kcal/day), alcohol consumption (g/day), adherence to a specific dietary regimen at baseline (yes/no), intake of ultra-processed foods (servings/day), and dietary sodium and potassium intake (mg/day). The model also accounted for family history of hypertension, baseline prevalence of hypercholesterolemia, hypertriglyceridemia, and depression, as well as analgesic medication use. Analyses were stratified by deciles of age and by year of entry into the cohort.

**Table 3:** Adjusted Hazard ratio ^a^ (95% confidence interval) for incident hypertension according to categories of underestimation and overestimation by sex.

	Adequate	Underestimation Obesity (R)-Overweight (P)	Underestimation Overweight (R)-Normal (P)	Underestimation Normal (R)-Underweight (P)
**Men**				
Cases/person-years	921/50,234	110/2473	135/6470	14/886
Incident rate 10^−3^ years^−1^ (95%CI)	18.3 (17.1–19.5)	45.2 (37.4–54.6)	21 (17.6–24.7)	15.8 (9.4–26.7)
Age-adjusted model	Reference	1.95 (1.59–2.38) *	1.02 (0.85–1.22)	0.85 (0.50–1.45)
Multivariate-adjusted model ^a^	Reference	1.17 (0.94–1.46)	0.99 (0.82–1.18)	1.19 (0.69–2.02)
**Women**				
Cases/person-years	799/105,231	35/1653	68/4208	7/469
Incident rate 10^−3^ years^−1^ (95%CI)	7.6 (7.1–8.1)	21.1 (15.2–29.4)	15.9 (12.5–20.2)	16.8 (8.0–35.3)
Age-adjusted model	Reference	2.31 (1.64–3.26) *	1.70 (1.32–2.18) *	2.11 (1.003–4.46) *
Multivariate-adjusted model ^a^	Reference	0.99 (0.69–1.43)	1.29 (1.02–1.66) *	2.89 (1.36–6.13) *
	Adequate	Overestimation Underweight (R)-Normal(P)	Overestimation Normal (R)-Overweight(P)	
**Men**				
Cases/person-years	921/50,234	1/186	143/7309	
Incident rate 10^−3^ years^−1^ (95%CI)	18.3 (17.1–19.5)	6.1 (2.9–14.7)	19.4 (16.2–22.6)	
Age-adjusted model	Reference	0.41 (0.05–2.89)	0.94 (0.79–1.11)	
Multivariate-adjusted model ^a^	Reference	0.62 (0.09–4.45)	0.99 (0.83–1.19)	
**Women**				
Cases/person-years	799/105,231	28/8041	91/9011	
Incident rate 10^−3^ years^−1^ (95%CI)	7.6 (7.1–8.1)	3.5 (2.9–4.2)	10 (7.1–12.1)	
Age-adjusted model	Reference	0.61 (0.42–0.89)	1.008 (0.81–1.26)	
Multivariate-adjusted model ^a^	Reference	0.94 (0.64–1.38)	0.88 (0.71–1.10)	

* *p* < 0.005. R: Nutritional status by BMI P: Nutritional Status perceived by Stunkard’s Figure. ^a^ The main model was adjusted for several potential confounders, including age, sex, smoking status (categorized as never, current, or former), physical activity levels (measured in MET-hours per week), and participants’ average weight throughout the follow-up period. Additional adjustments included adherence to the Mediterranean diet (MedDiet score), total energy intake (kcal/day), alcohol consumption (g/day), adherence to a specific dietary regimen at baseline (yes/no), intake of ultra-processed foods (servings/day), and dietary sodium and potassium intake (mg/day). The model also accounted for family history of hypertension, baseline prevalence of hypercholesterolemia, hypertriglyceridemia, and depression, as well as analgesic medication use. Analyses were stratified by deciles of age and by year of entry into the cohort.

**Table 4:** Sensitivity analyses: HR and 95% CI (Underestimation vs. adequate perception) for incident hypertension.

	Men			Women		
	HR	95% CI	Cases/*n*	HR	95% CI	Cases/*n*
1. Main analysis	1.06	0.92–1.23	1324/67,558	1.25	1.01–1.55	1028/128,613
2. Excluding prevalent hypertriglyceridemia	1.15	0.98–1.35	1118/62,258	1.32	1.05–1.65	989/125,821
3. Excluding prevalent hypercholesterolemia	1.14	0.96–1.35	958/55,672	1.21	0.95–1.54	863/114,986
4. Excluding extreme energy limits (<p1 or >p99)	1.06	0.91–1.22	1307/66,651	1.27	1.02–1.58	1002/126,581
5. Excluding baseline depression	1.04	0.90–1.20	1290/65,601	1.23	0.99–1.55	982/122,256
6. Excluding early incident hypertension	1.04	0.90–1.25	1048/66,997	1.28	1.003–1.63	845/128,243
7. Censuring follow-up ≥14 years	1.08	0.93–1.25	1160/59,148	1.21	0.95–1.53	857/112,657
8. Adjusting for baseline BMI	0.93	0.80–1.08	1324/67,558	1.08	0.85–1.36	1028/128,613

1. The main adjusted model included covariates such as age, sex, smoking status (never, current, or former), physical activity (in MET-hours per week), average weight during the follow-up period, adherence to the Mediterranean diet (MedDiet score), total energy intake (kcal/day), alcohol consumption (g/day), adherence to a specific diet, intake of ultra-processed foods (servings/day), dietary intake of sodium and potassium, family history of hypertension, baseline prevalence of hypercholesterolemia, hypertriglyceridemia, depression, and the use of analgesic medications. The model was also stratified by age deciles and year of cohort entry. 2. Sensitivity analysis excluding the adjustment for prevalent hypertriglyceridemia. 3. Sensitivity analysis excluding the adjustment for prevalent hypercholesterolemia. 4. Exclusion of participants with extreme daily energy intake (<p1 or >p99). 5. Exclusion of participants with baseline depression. 6. Exclusion of participants diagnosed with hypertension within the first two years of follow-up. 7. Exclusion of participants with a follow-up period equal to or exceeding 14 years. 8. Model additionally adjusted for baseline BMI.

## Data Availability

The data from the SUN Project that support our findings are available upon request from the Department of Preventive Medicine and Public Health, School of Medicine, University of Navarra (Spain) at sun@unav.es.

## Abbreviations

The following abbreviations are used in this manuscript:

FFQ	Food Frequency Questionnaire
BMI	Body Mass Index
FRS	Stunkard’s Figure Rating Scale
CVD	Cardiovascular diseases
